# Current ciprofloxacin usage in children hospitalized in a referral hospital in Paris

**DOI:** 10.1186/1471-2334-13-245

**Published:** 2013-05-27

**Authors:** Zhi-Tao Yang, Jean-Ralph Zahar, Fréderic Méchaï, Martine Postaire, Stéphane Blanot, Sarah Balfagon-Viel, Xavier Nassif, Olivier Lortholary

**Affiliations:** 1 Service des Maladies Infectieuses et Tropicales & Centre d’Infectiologie Necker-Pasteur, Université Paris Descartes, Hôpital Necker-Enfants Malades, Paris, France; 2 Equipe mobile d’infectiologie, Hôpital Necker-Enfants Malades, Paris, France; 3 Service Microbiologie–Hygiène Hospitalière, Hôpital Necker-Enfants Malades, Paris, France; 4 Service Pharmacie, Hôpital Necker-Enfants malades, Paris, France; 5 Service d’Anesthésie, Hôpital Necker-Enfants malades, Paris, France; 6 First author: Emergency department, Pôle Sino-Français de Recherche en Science du Vivant et Génomique, Ruijin Hospital in Shanghai, No 197, Ruijin Er Road, Shanghai 200025, China

**Keywords:** Ciprofloxacin, Children, Cystic fibrosis, Appropriate prescription

## Abstract

****Background**:**

Fluoroquinolones are used with increasing frequency in children with a major risk of increasing the emergence of FQ resistance. FQ use has expanded off-label for primary antibacterial prophylaxis or treatment of infections in immune-compromised children and life-threatening multi-resistant bacteria infections. Here we assessed the prescriptions of ciprofloxacin in a pediatric cohort and their appropriateness.

**Methods:**

A monocenter audit of ciprofloxacin prescription was conducted for six months in a University hospital in Paris. Infected site, bacteriological findings and indication, were evaluated in children receiving ciprofloxacin in hospital independently by 3 infectious diseases consultants and 1 hospital pharmacist.

**Results:**

Ninety-eight ciprofloxacin prescriptions in children, among which 52 (53.1%) were oral and 46 (46.9%) parenteral, were collected. 45 children had an underlying condition, cystic fibrosis (CF) (21) or an innate or acquired immune deficiency (24). Among CF patients, the most frequent indication was a broncho-pulmonary *Pseudomonas aeruginosa* infection (20). In non-CF patient, the major indications were broncho-pulmonary (25), urinary (8), intra-abdominal (7), operative site infection (5) and bloodstream/catheter (2/4) infection. 62.2% were microbiologically documented. Twenty-three (23.4%) were considered “mandatory”, 48 (49.0%) “alternative” and 27 (27.6%) “unjustified”.

**Conclusion:**

In our university hospital, only 23.4% of fluoroquinolones prescriptions were mandatory in children, especially in *Pseudomonas aeruginosa* healthcare associated infection. Looking to the ecological risk of fluoroquinolones and the increase consumption in children population we think that a control program should be developed to control FQ use in children. It could be done with the help of an antimicrobial stewardship team.

## Background

Fluoroquinolones (FQ) are licensed and widely indicated for use in adults, owing to the agents’ broad-spectrum antibacterial activity, their extensive tissue and intracellular penetration, and their suitability for oral administration. However, FQ use in pediatric patients has been contraindicated by regulatory authorities in the United States and the European Union, given the cartilage damage that they may induce in juvenile animal models. Nonetheless, FQ use in pediatric patients has increased as shown in the United States, where approximately 520,000 prescriptions were written for children and adolescents younger than 18 years in 2002 [1]. The safety of fluoroquinolones is questioned in pediatric population [2]. Joint disorder and arthromyalgia could be as frequent as 8.3% [3-5]. In pediatric cystic fibrosis patients with acute pulmonary exacerbation caused by *P. aeruginosa*[6], ciprofloxacin is initially administered intravenously at a dose of 30 mg/kg/day divided every 8 h, followed by an oral administration at 40 mg/kg/day divided every 12 h [7]. Simultaneously, FQ use has expanded off-label for primary antibacterial prophylaxis or treatment of infections in immune-compromised patients, infections due to life-threatening multi-resistant bacteria, or salmonellosis or shigellosis and cholangitis [1,6,8,9]. In 2004, ciprofloxacin became the first fluoroquinolone agent approved by United States Food and Drug Administration for use in children 1 through 17 years of age [10]. As suggested by several studies [11-13] the increased use of fluoroquinolones will subsequently contribute to the spread of resistance.

The aim of this observational study was to evaluate the prescription of ciprofloxacin for pediatric infections.

## Methods

A mono-center audit of ciprofloxacin prescription was conducted between 1st December 2007 and 31st May 2008 at Necker-Enfants malades University hospital in Paris. All consecutive pediatric patients (age <18 years) admitted in our hospital, with fluoroquinolone prescribed in hospital were included in this study. All prescriptions were identified by pharmacy’s computer. Descriptive data concerning patients’ characteristics (age, underlying disease, current status, immune status such as neutropenia <500/mm^3^ or immunosuppressive agent use such as receiving 1 mg/kg/d prednisone for more than 1 weeks or equivalent), the current ciprofloxacin regimen (drug, indication and bacteriologic findings, justification for use, concomitant drugs administered) were collected from patients’ medical documents.

Nosocomial infection was defined as an episode of infection from patients who had been hospitalized for 48 hours or longer while health care-associated infection was defined as from a patient at the time of hospital admission or within 48 hours of admission if the patient fulfilled any of the following criteria (hemodialysis, intravenous chemotherapy during the past 30 days, hospitalization for at least two days during the past 90 days, home intravenous therapy or wound care during the past 30 days). Community-acquired infection was defined as an episode of infection at the time of hospital admission or within the 48 hours after hospital admission for patients who did not fit the criteria for a health care-associated infection [14].

The short-term side effects, such as nausea, abdominal pain, diarrhea, vomiting, headache, rash or photosensitivity, arthralgia and myalgia, were reported by patient’s physician during the whole hospitalization and reviewed twice weekly by our mobile infectious diseases team (1 resident, 2 senior physicians and 1 professor in infectious diseases).

Appropriateness of ciprofloxacin prescription was retrospective performed separately by three experts in infectious diseases and one hospital pharmacist. Prescriptions were categorized as “mandatory, alternative or unjustified”. Ciprofloxacin use was considered “mandatory” if susceptibility testing showed resistance to all beta-lactams and TMP-SMX; susceptible only to FQ, or aminoglycoside or colistin, or the ciprofloxacin was the only oral therapy available.

Prescriptions were considered “alternative” in the setting of ciprofloxacin use being indicated by clinical situation or susceptibility testing, in the presence of an available alternative enteral or parenteral antibiotic choice. Finally “unjustified” prescriptions were defined as ciprofloxacin prescription not indicated according to the clinical situation, and/or susceptibility results. The latter two groups defined the inappropriate fluoroquinolone usage.

For the discharged children, the follow-up and monitoring was performed by their respective physician and adverse events were reported to our mobile team if myalgia or arthralgia was mentioned by patients.

Data protection was approved by the Institutional Review Board of Necker-Enfants Malades Hospital. No ethical committee approval was required under French regulations.

## Results

A total of 11,268 children were admitted between 1st December 2007 and 31st May 2008 at the Necker-Enfants malades hospital in Paris. 98 new ciprofloxacin prescriptions were collected. Prescriptions mainly originated from general pediatric wards (22.4%), immuno-hematology department (20.4%), polyvalent intensive care units (12.2%), cardiac surgery unit (11.2%), general surgery (9.2%) and orthopedic surgery (9.2%). Twenty-one patients with cystic fibrosis and 77 without CF received at least one dose of ciprofloxacin during the study period.

### Demographic characteristics and underlying diseases of the population

The median age of the 98 children receiving ciprofloxacin was 10 years (1 month – 15 years). 35 were younger than 2 years, 17 were ≥2 and <6 years old, and 46 were ≥6 years up to puberty onset. The gender ratio was 1.3 (M/F). 45/98 (45.9%) had an underlying condition, the most frequent being cystic fibrosis (n = 21, 21.4%), followed by immune deficiencies such as neutropenia (<500/mm^3^) or severe combined immunodeficiency (SCID) (12.2%) or use of any immunosuppressive agent (12.2%) (corticosteroids, cyclosporine or tacrolimus) because of solid organ transplant (3 hepatic, 2 intestinal and hepatic, 1 intestinal, 1 renal and 1 cardiac) and 4 others (glycogenosis, hemophagocytosis syndrome, osteopetrosis and Wegener’s granulomatosis).

### Type of infections

Sixty-two infections were nosocomially-acquired, twenty-four were healthcare-associated and thirty-five were community acquired. The remaining child received ciprofloxacin for prophylaxis during orthopedic surgery (Table 1).

**Table 1:** Infections sites in 97 children receiving ciprofloxacin

	**Community-acquired infection**	**Healthcare-associated infection**	**Nosocomial infection**
Infection site			
Broncho-pulmonary n (%)	5 (45.4)	17 (70.8)	24 (38.6)
Urinary tract n (%)	2 (18.2)	2 (8.3)	4 (6.5)
Bloodstream/catheter infection n (%)	0 (0)	0 (0)	2(3.2) /4 (6.5)
Infection of operative site n (%)	0 (0)	0 (0)	5 (8.2)
Bone or joint n (%)	2 (18.2)	0 (0)	2 (3.2)
Intra-abdominal n (%)	1 (9.1)	0 (0)	6 (9.7)
Mediastinal^$^ n (%)	0 (0)	0 (0)	3 (4.8)
Cerebral n (%)	1 (9.1)	1 (4.2)	2 (3.2)
Endocarditis n (%)	0 (0)	0 (0)	2 (3.2)
Others n (%)	0 (0)	4^£^ (16.7)	8^§^ (12.9)
Total n (%)	11 (100)	24 (100)	62 (100)

^£^: 2 BCGitis and ocular infection and cutaneous infection each.

^§^: Fever in 8 immunocompromised children among whom 4 with current neutropenia.

Among 98 children who received ciprofloxacin, one received it as prophylaxis during orthopedic surgery (not shown in Table 1).

^$^: Mediastinal infection means mediastinitis after cardiac or thoracic surgery.

Table 2 reports the site of infections and the bacteriological findings. In CF patients, the most frequent clinical indications were *P. aeruginosa* (95.2%) or methicillin-resistant *S. aureus* broncho-pulmonary infection (4.8%). In non-CF patients, *P. aeruginosa* infections represented 16.9% of the ciprofloxacin prescriptions.

**Table 2:** Bacteriological findings and indication of ciprofloxacin in 98 children

	**Patients without CF (n = 77)**	**Patients with CF (n = 21)**
Bacteriological finding n (%)	40 (51.9)	21 (100)
Presence of *P. aeruginosa* n (%)	13 (16.9)	20 (95.2)
Mixed bacterial infection n (%)	3 (3.9)	1 (4.8)
Antibiotic combination n (%)	65 (84.4)	21 (100)
Indication for ciprofloxacin		
Active infection n (%)	76 (98.7)	21 (100)
Bronchopulmonary n (%)	25 (32.4)	21 (100)
Urinary tract n (%)	8 (10.4)	0
Intra-abdominal infection n (%)	7 (9.1)	0
Bloodstream infection/catheter infection n (%)	2 (2.6)/4 (5.2)	0
Operative site infection n (%)	5 (6.5)	0
Bone or joint n (%)	4 (5.2)	0
Cerebral n (%)	4 (5.2)	0
Mediastinitis^$^ n (%)	3 (3.9)	0
Endocarditis n (%)	2 (2.6)	0
Miscellaneous^§^ n (%)	12 (15.6)	0
Prophylaxis n (%)	1 (1.3)	0

Miscellaneous^§^:2 BCGitis, 1 Ocular infection, 1 cutaneous infection and 8 fever in immunocompromised children among whom 4 with current neutropenia.

^$^: Mediastinal infection means mediastinitis after cardiac or thoracic surgery.

### Adequacy of ciprofloxacin prescriptions

The median ciprofloxacin’s dosage (Table 3) was 40 mg/kg/24 h (QR range 30–40 mg/kg/24 h), and 20 mg/kg/24 h (QR range was 20–30 mg/kg/24 h), in CF and non-CF patients, respectively.

**Table 3:** Ciprofloxacin dosages in 98 children

			**Dose (mg/kg/day)**				**Frequency of administration**			Adequate dose* n (%)
		**n**	**Min**	**Max**	**Median**	**Mean (SD)**	**qd**	**b.i.d**	**t.i.d**	
Patients without CF	oral cipro	32	15	50	20	23(7)	1	30	1	31 (96.9)
	i.v. cipro	45	10	60	20	23(9)	1	36	8	38 (84..4)
	Total	77	10	60	20	23(8)	2	66	9	69 (89.6)
Patients with CF	oral cipro	20	30	50	40	37(5)	0	19	1	13 (61.9)
	i.v. cipro	1	/	/	/	/	0	1	0	1 (100)
	Total	21	30	50	40	37(5)	0	20	1	14 (66.7)
Total		98	10	60	20	26(9)	2	86	10	83 (84.7)

* Adequate dose in patients without CF was considered as 6 to 10 mg/kg/dose intravenous t.i.d. or 10 to 15 mg/kg/dose orally b.i.d. in children with CF, 10 or 15 mg/kg/dose intravenous three times daily or twice daily followed by 20 mg/kg/dose per os twice daily.

Among the 98 ciprofloxacin prescriptions, 23 (23.4%) were considered as “mandatory”, 48 (49.0%) were “alternative” and 27 (27.6%) were “unjustified” (Table 4).

**Table 4:** Description of mandatory ciprofloxacin prescriptions

		**Mandatory cipro prescription n (%)**
With bacteriological finding		23 (100)
	*P. aeruginosa*	23
With cystic fibrosis		17
Type of infection		
	Community-acquired	1
	Healthcare associated	13
	Nosocomially-acquired	9
Justified by		
	Only cipro susceptible	12
	Cipro per os	11
Site of infection		
	Pulmonary	19 (82.6)
	Bone	2
	Skin	1
	Cerebral	1
Total		23 (100)

Among 23 prescriptions classified as mandatory, 17 children had CF and 6 not. All had microbiologically documentation with *P. aeruginosa* (Table 4).

Forty-eight prescriptions were classified as “alternative”. Among them, 28 had culture results available (6 due to *P. aeruginosa* and 22 due to other bacteria) and 20 without. All could have received an alternative antibiotic (Table 5).

**Table 5:** Description of alternative ciprofloxacin prescriptions

			**Alternative cipro prescription n (%)**
With bacteriological finding			28 (58.3)
	*P. aeruginosa*		6
		Cystic fibrosis pulmonary infection	3
		Site of infection for with *P. aeruginosa* without CF#	3
		Oral cipro	6
	*E. coli*		6
	CNS ¶		5
	*K. pneumoniae*		3
	MRSA £		2
	*S. marcescens*		2
	Others *		4
		Oral cipro	9
		Intravenous cipro	13
		Site of infection for non *P. aeruginosa*$	22
Alternative by another active antibiotic			28
Without bacteriological finding			20 (41.7)
Alternative by another antibiotic			20
	Site of infection for non bacteriologically documented infection &		17
	Empiric therapy for fever with current neutropenia		3
		Intravenous cipro	13
		Oral cipro	7
Total			48 (100)

CNS = coagulase negative *Staphylococcus*, MRSA = Methicillin-resistant *S. aureus.*

¶ 5 CNSs were isolated from hemocultures, of which 2 had endocarditis, 1 catheter related bloodstream infection.

# 2 urinary infection and 1 operative site infection with *P. aeruginosa.*

£ One child with CF had MRSA pulmonary infection.

* *E. cloacae*, *H. influenzae*, *M. catarrhalis*, *K. oxytoca*, each.

$ 8 pulmonary, 3 urinary, 3 intra-abdominal, 2 bloodstream, 3 operative site infection and 1 endocarditis, catheter related infection, osteomyelitis each.

& 5 pulmonary, 3 meningitis, 2 mediastinitis, 2 BCGitis and intra-abdominal infection, endocarditis, post-trauma ocular infection, osteomyelitis, urinary each.

27 were classified as “unjustified” and corresponded to any ciprofloxacin prescription that was not indicated according to the clinical situation and/or microbiological results. Among these 27, 10 had culture results available, 4 due to *P. aeruginosa* susceptible to all known active antibiotics and 6 due to another microorganism. All of these 10 unjustified prescriptions (5 orally and 5 intravenously) were not indicated based on the current clinical situation or microbiological results (Table 6).

**Table 6:** Description of unjustified ciprofloxacin prescriptions

			**Unjustified cipro prescription n (%)**
With bacteriological finding			10 (37.0)
	*P. aeruginosa*		4
		Intravenous cipro	4
		Site of infection for *P. aeruginosa*#	4
	Multiply susceptible *E. coli*		1
	ESBL producing *E. coli*		1
	CNS		4
		Intravenous cipro	1
		Oral cipro	5
		Site for non-*P. aeruginosa* infections$	
Unjustified by antibiogram results			6
Unjustified by intravenous cipro in susceptible *P. aeruginosa* infection			4
Without bacteriological finding			17 (63.0)
	Site of non bacteriologically-documented infection		12
		Pulmonary	7
		Abdominal	2
		Catheter related infection	1
		Operative site infection	1
		Prophylaxis	1
	Empiric therapy for fever		5
		Neutropenia	1
		Immunocompromised but without Neutropenia	4
Unjustified by clinical situation			17
Total			27 (100)

# 2 pulmonary and 2 catheter related infection.

$ 2 pulmonary, 2 urinary and intra-abdominal, mediastinitis each.

### Side effect of ciprofloxacin

We did not notice any bone/joint short-term side effects among the 98 children, even 2 weeks after the end of ciprofloxacin therapy.

## Discussion

In our study, 76.6% of ciprofloxacin indications were inappropriate, most often (49.0%) because another antibacterial could have been considered and 27.6% did not correspond to validated indications. The American Academy of Pediatrics [6] supports the use of ciprofloxacin in pediatric cystic fibrosis patients with acute pulmonary exacerbation associated with *P. aeruginosa* infection, while in recent years, recommendations have also included *P. aeruginosa* osteochondritis, shigellosis, salmonellosis, and *C. jejuni* infections, prophylaxis during neutropenia, empiric therapy in febrile neutropenic children with cancer, treatment of patients with multidrug-resistant Gram-negative bacteremia or meningitis, and combination use with other agents to treat multidrug-resistant mycobacterial disease. Although a recent study showed that levofloxacin may represent an effective therapy [15], the American Academy of Pediatrics does not recommend it for first-line therapy of respiratory tract infection in children [10]. The experience of use FQ in fever with neutropenia or in critically ill children has been limited. Sideri’s study [16], including 18 critically ill children, showed that ciprofloxacin might be a useful option for critically ill children without CF. More recently, Sung L *et al.*[17] has meta-analyzed 740 low-risk fever with neutropenia episodes in 10 studies, which showed 17% to 24% treatment failure with ciprofloxacin monotherapy or FQ combination therapy, but no cases of infectious deaths has been reported and the rates of adverse events were very low. But in our study, we still considered 4 ciprofloxacin combination prescriptions for fever and neutropenia as “unjustified”.

The use of an incorrect dosage was also frequent among children treated with ciprofloxacin. Indeed, in our study, 28 (28.6%) received an incorrect ciprofloxacin dosage. Furthermore, of the 23 pediatric patients in whom ciprofloxacin was prescribed as mandatory, 9 (39.1%) received an incorrect dose (4 received an excessive dose while 5 an insufficient dose). Sermet-Gaudelus *et al.* suggested that an excessive daily dosage was considered when it was more than 40 mg/kg in non CF patient and an insufficient dosage was considered when it was less than 40 mg/kg/d in CF patients and less than 20 mg/kg/d in non CF patients [18]. The likelihood of FQ drug toxicity may also be affected by inappropriate FQ prescribing. In fact, higher dose and longer duration of FQ therapy have both been associated with a greater risk of adverse events, including emergence of multidrug resistance [19].

The use of FQ has been limited in pediatric infection due to their potential side-effects which have been found to affect cartilage in juvenile animals, resulting in arthropathy [1]. However, in recently controlled, retrospective studies, major arthropathy (as observed in animals) has never been notified in children [4,5]. In addition, if a musculoskeletal event did occur in the exposed children, it was always minor and always reversible. In recent years, FQ use in children has increased in the United States, but not in France [3]. Few studies have evaluated the appropriate use of FQ prescriptions in children in order to avoid the side effects and their ecological impact [20]. In our study, we did not notice any bone/joint short-term side effects among the 98 children, even 2 weeks after the end of ciprofloxacin therapy, because our observational period was too short or the cases were too small to evaluate the side effects of ciprofloxacin in children use.

Our findings suggest that our current patterns of FQ use can be greatly improved in children in an effort to reduce the spread of FQ resistance. Recently, several studies have showed that about 50%, even up to 70%, of antibiotic usage in hospitals and in emergency departments were inappropriate [21-24], in accordance with our results.

The assistance of a medical microbiologist/infectious diseases specialist in addition to educational presentations as done in adult’s populations have led to 3 to 4-fold sustained reduction of inappropriate prescriptions, but also to a significant improvement in quality of ciprofloxacin prescriptions [25] in accordance with existing guidelines [22].

The most important situation where we need FQ in children is cystic fibrosis and *P. aeruginosa* infection. Other indications are rare and require individual careful assessment. The limitations of our study were that it was an observational monocentric study and was done within a short period of time. A larger study would have indeed provided more meaningful information. The duration of FQ use was not collected in our study.

## Conclusion

In our university hospital, only 23.4% of fluoroquinolones prescriptions were mandatory in children, especially for *P. aeruginosa* healthcare associated infection. Looking to the ecological risk of fluoroquinolones and the increase consumption in children population, we think that a control program should be developed to control FQ use in children. It could be done with the help of an antimicrobial stewardship team.

## Consent

Written informed consent was obtained from the patient for publication of this report and any accompanying images.

## Competing interests

The authors declare that they have no competing interests.

## Authors’ contributions

ZTY, JRZ, MP, SB and XN made substantial contributions to conception and design. ZTY, JRZ, FM and SBV participated in acquisition of data. ZTY, JRZ and OL drafted the manuscript. JRZ and OL revised it critically. All authors read and approved the final manuscript.

## Pre-publication history

The pre-publication history for this paper can be accessed here:

http://www.biomedcentral.com/1471-2334/13/245/prepub
